# Associations among Lead Dose Biomarkers, Uric Acid, and Renal Function in Korean Lead Workers

**DOI:** 10.1289/ehp.7317

**Published:** 2004-09-30

**Authors:** Virginia M. Weaver, Bernard G. Jaar, Brian S. Schwartz, Andrew C. Todd, Kyu-Dong Ahn, Sung-Soo Lee, Jiayu Wen, Patrick J. Parsons, Byung-Kook Lee

**Affiliations:** ^1^Division of Occupational and Environmental Health, Department of Environmental Health Sciences, Johns Hopkins UniversityBloomberg School of Public Health, Baltimore, Maryland, USA; ^2^Department of Medicine, Johns Hopkins University School of Medicine, Baltimore, Maryland, USA; ^3^Department of Epidemiology, Johns Hopkins University Bloomberg School of Public Health, Baltimore, Maryland, USA; ^4^Department of Community and Preventive Medicine, Mount Sinai School of Medicine, New York, New York, USA; ^5^Institute of Industrial Medicine, SoonChunHyang University, Asan, South Korea; ^6^Lead Poisoning/Trace Elements Laboratory, Wadsworth Center, New York State Department of Health, Albany, New York, USA

**Keywords:** kidney function, mechanisms, occupational lead exposure, renal early biologic effect markers, uric acid

## Abstract

Recent research suggests that both uric acid and lead may be nephrotoxic at lower levels than previously recognized. We analyzed data from 803 current and former lead workers to determine whether lead biomarkers were associated with uric acid and whether previously reported associations between lead dose and renal outcomes were altered after adjustment for uric acid. Outcomes included uric acid, blood urea nitrogen, serum creatinine, measured and calculated creatinine clearances, and urinary *N*-acetyl-β-d-glucosaminidase (NAG) and retinol-binding protein. Mean (± SD) uric acid, tibia lead, and blood lead levels were 4.8 ± 1.2 mg/dL, 37.2 ± 40.4 μg/g bone mineral, and 32.0 ± 15.0 μg/dL, respectively. None of the lead measures (tibia, blood, and dimercaptosuccinic-acid–chelatable lead) was associated with uric acid, after adjustment for age, sex, body mass index, and alcohol use. However, when we examined effect modification by age on these relations, both blood and tibia lead were significantly associated (β= 0.0111, *p* < 0.01 and β= 0.0036, *p* = 0.04, respectively) in participants in the oldest age tertile. These associations decreased after adjustment for blood pressure and renal function, although blood lead remained significantly associated with uric acid (β= 0.0156, *p* = 0.01) when the population was restricted to the oldest tertile of workers with serum creatinine greater than the median (0.86 mg/dL). Next, in models of renal function in all workers, uric acid was significantly (*p* < 0.05) associated with all renal outcomes except NAG. Finally, in the oldest tertile of workers, associations between lead dose and NAG were unchanged, but fewer associations between the lead biomarkers and the clinical renal outcomes remained significant (*p* ≤0.05) after adjustment for uric acid. In conclusion, our data suggest that older workers comprise a susceptible population for increased uric acid due to lead. Uric acid may be one, but not the only, mechanism for lead-related nephrotoxicity.

Historically, gout was common among patients with lead poisoning (Batuman 1993). More recently, associations between various measures of lead dose and serum uric acid (urate) levels have been reported in studies of occupationally exposed populations (Ehrlich et al. 1998; Wang et al. 2002) as well as in general population studies (Lin et al. 2002; Shadick et al. 2000). These associations are present at much lower lead doses than those associated with gout in historical lead poisoning. Lead exposure also increases the risk for adverse renal outcomes. Lead has been reported to cause nephrotoxicity by several mechanisms, although it is not known which of these is the predominant pathway (Nolan and Shaikh 1992; Sanchez-Fructuoso et al. 2002; Vaziri 2002). Uric acid is also a nephrotoxicant, and increasing evidence suggests that this toxicity occurs at lower levels than previously recognized (Johnson et al. 2003). Several adverse renal and vascular outcomes have been reported in a recently developed rodent model of low-level hyperuricemia, including hypertension and tubulointerstitial fibrosis (Mazzali et al. 2001a), renal afferent arteriolopathy (Mazzali et al. 2002), glomerular hypertrophy, glomerulosclerosis (Nakagawa et al. 2003), and glomerular hypertension (Sanchez-Lozada et al. 2002). More important, uric acid in this model accelerates renal dysfunction from other causes (Kang et al. 2002; Mazzali et al. 2001b). This raises the intriguing possibility that increased uric acid is one mechanism by which lead causes nephrotoxicity.

In our recently reported analyses of data from the first of three evaluations in a longitudinal study of the health effects of inorganic lead exposure in 803 current and former lead workers (Weaver et al. 2003), we found associations between lead exposure and dose measures and adverse renal function outcomes. Lead measures were associated with decreased renal function, primarily in the oldest tertile of workers (> 46 years of age). Therefore, we analyzed data from the entire population of lead workers and conducted separate analyses of the oldest tertile of workers in some models to determine whether the lead biomarkers were associated with uric acid and whether uric acid levels were associated with renal function outcomes. In addition, we evaluated whether relations between the lead biomarkers and renal outcomes were altered after adjustment for uric acid.

## Materials and Methods

### Study overview and design.

We report data from 803 current and former lead workers who completed the first of three annual evaluations in a longitudinal study of the renal, vascular, hematopoietic, and nervous system effects of inorganic lead exposure. Participants were evaluated between 24 October 1997 and 19 August 1999. All participants provided written, informed consent. The study protocol was approved by institutional review boards at the SoonChunHyang University and the Johns Hopkins University Bloomberg School of Public Health. Participation in the study was voluntary, and workers were paid approximately $30 for their time and effort.

### Study population.

As previously described (Schwartz et al. 2001; Weaver et al. 2003), workers were recruited from 26 different plants that produced lead batteries, lead oxide, lead crystal, or radiators or were secondary lead smelters. Workers were designated as lead workers based on the potential for exposure to lead in the manufacturing process. No medical exclusionary criteria were used. Study participants were not currently occupationally exposed to other known renal toxicants.

### Data collection.

Data collection was completed either at the Institute of Industrial Medicine of the SoonChunHyang University in Chonan or at the study plants, using previously reported methods (Schwartz et al. 2001; Weaver et al. 2003). Data and biologic specimens collected included a standardized questionnaire on demographics, medical history, and occupational history; blood pressure measured with a Hawksley random zero sphygmomanometer (Lee et al. 2001); height and weight measurement; a blood specimen [for blood lead, blood urea nitrogen (BUN), serum creatinine, and uric acid]; a spot urine sample [for *N*-acetyl-β-d-glucosaminidase (NAG), retinol-binding protein (RBP), and creatinine]; and tibia lead concentration. A 4-hr urine collection after oral administration of 10 mg/kg dimercaptosuccinic acid (DMSA) was also obtained to measure DMSA chelatable lead and creatinine clearance (787 participants completed this collection).

### Laboratory methods.

The lead biomarkers and renal outcomes were measured using previously reported assays (Schwartz et al. 2001; Weaver et al. 2003). In brief, blood lead was measured (Fernandez 1975) with an Hitachi 8100 Zeeman background-corrected atomic absorption spectrophotometer (Hitachi Ltd. Instruments, Tokyo, Japan) at the Institute of Industrial Medicine, a certified reference laboratory for lead in South Korea. Tibia lead was assessed via a 30-min measurement of the left mid-tibia diaphysis using ^109^Cd in a back-scatter geometry to fluoresce the K-shell X rays of lead. The lead X rays were recorded with a radiation detector and then quantified and compared with calibration data to estimate the concentration of lead in bone (Todd and Chettle 1994; Todd and McNeill 1993). The emitted K-shell X rays were attenuated as they passed through bone and overlying tissues. The lead X rays were therefore normalized to the amount of elastic scattering from the bone itself to yield a measurement accuracy that is independent of the distance between the radiation source and the subject, subject positioning, small subject movements, overlying tissue thickness, and bone size, shape, geometry, and density (Todd 2000a, 2000b; Todd and Chettle 1994; Todd and McNeill 1993). All point estimates, including negative values, were retained in the statistical analyses in order to minimize bias and to avoid censoring of data (Kim et al. 1995). Urine lead levels in the 4-hr collection were measured at the Wadsworth Center of the New York State Department of Health (Albany, NY, USA) by electrothermal atomic absorption spectrometry with Zeeman background correction (model 4100ZL; Perkin Elmer, Norwalk, CT, USA) (Parsons and Slavin 1999). BUN, serum creatinine, and uric acid were measured via an automatic chemical analyzer (model TBA 40FR Biochemical Analyzer; Toshiba, Tokyo, Japan). Urine creatinine was measured in spot samples (for adjustment of NAG and RBP) and in the 4-hr sample after DMSA (for determination of measured creatinine clearance and adjustment of DMSA-chelatable lead levels), using a modification of the Sigma kit (Sigma Chemical Company, St. Louis, MO, USA) assay (Weaver et al. 2000). Measured creatinine clearance was defined as [(urinary creatinine in milligrams per deciliter × urine volume in milliliters) ÷ serum creatinine in milligrams per deciliter] ÷ collection time in minutes. Calculated creatinine clearance was obtained from the Cockcroft-Gault equation (Cockcroft and Gault 1976). NAG activity (expressed in micromoles of substrate converted per hour) was measured using the PPR NAG test kit (PPR Diagnostics, Ltd., London, UK), and RBP was measured using a modification of the method of Topping et al. (1986). As previously reported by Weaver et al. (2003), the mean between-day coefficient of variation (CV) for 138 random NAG samples assayed in duplicate was 6.0%; the CV for RBP was 7.4% (75 samples assayed in duplicate).

### Statistical analysis.

**Figure 1:**
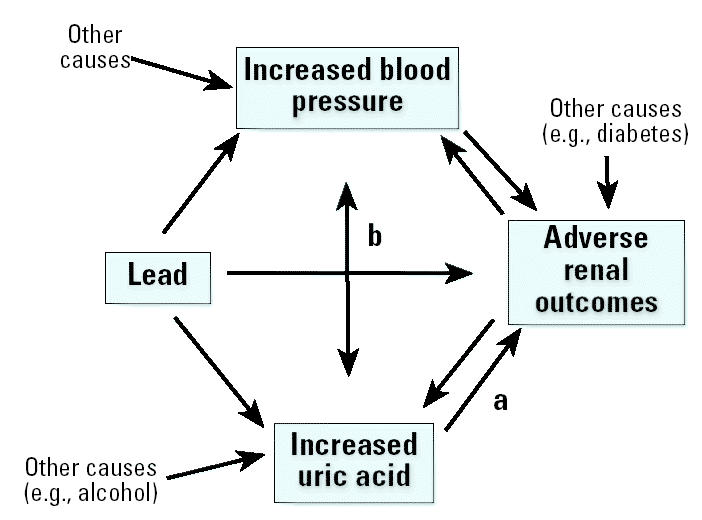
Biologic relations among lead, uric acid, blood pressure, and renal function variables. Uric acid is an established nephrotoxicant at high levels (a); the threshold for renal toxicity is uncertain. The association between uric acid levels and increased blood pressure may be causal or due to confounding (b). Specifically, high uric acid levels may cause hypertension secondary to renal dysfunction but whether low-level uric acid causes primary hypertension is less certain.

The overall goal of our analysis was to develop models that would allow hypotheses to be generated regarding causal pathways involving lead, uric acid, blood pressure, and renal function. As shown in Figure 1, these variables are biologically interrelated. As a result, adjustment for covariates presents unique challenges. Adjustment for renal function and blood pressure likely results in overcontrol when associations between lead measures and uric acid are being evaluated. This is because renal dysfunction and elevated blood pressure are risk factors for increased uric acid (Wortmann and Kelley 2001), and both can be caused or exacerbated by lead dose; thus, they may be in the causal pathway between lead and uric acid. On the other hand, because non-lead-related factors contribute to both renal dysfunction and elevated blood pressure, lack of adjustment for these variables in such models likely results in residual confounding. The interrelatedness of these variables, as it relates to the potential for confounding versus causality, has been extensively discussed in the literature pertaining to uric acid as a risk factor for adverse cardiac, vascular, and renal outcomes (Johnson et al. 2003). Therefore, we have presented our data both with and without additional adjustment.

**Figure 2:**
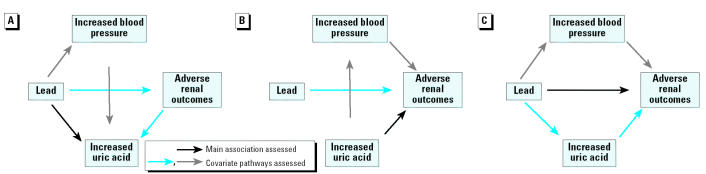
Biologic relations among variables in models from Tables 4–6. (*A*) Associations of lead biomarkers with uric acid (black arrow) in method 1 (Table 4). The gray arrows represent the blood pressure pathway added in method 2, Table 4; blue arrows represent the renal function pathway added in method 3 (Table 4). (*B*) Relations between uric acid levels and renal function outcomes. Data in Table 5 control for blood pressure (gray arrows); lead biomarkers (blue arrow) were also added to these methods (Table 6 shows selected methods in the oldest tertile of workers). (*C*) Associations of lead biomarkers, uric acid, and blood pressure with renal function outcomes (presented in Table 6). These methods specifically assessed the effect of uric acid (blue arrows) on the main association between lead biomarkers and renal outcomes (black arrow), while controlling for blood pressure (gray arrows) and other covariates.

Analysis in these current and former lead workers was directed toward the following steps: *a*) to evaluate associations of three lead dose biomarkers (tibia lead, blood lead, and DMSA-chelatable lead) with uric acid, with and without control for blood pressure and renal function, while controlling for other covariates (Figure 2A); *b*) to evaluate associations between uric acid and six renal function outcomes (BUN, serum creatinine, measured creatinine clearance, calculated creatinine clearance, RBP, and NAG), with and without control for lead, while adjusting for blood pressure and other covariates (Figure 2B); and *c*) to determine whether relations among these lead biomarkers and the six renal outcomes were altered by adjustment for uric acid, while controlling for other covariates, including blood pressure (Figure 2C). Statistical analysis was completed using SAS software (SAS Institute, Inc., Cary, NC, USA).

Initially, we examined variable distributions. The distributions of NAG and RBP showed departures from normality and were thus ln-transformed; the adequacy of this transformation was subsequently confirmed by examination of the residuals from regression models. Linear regression modeling was used to evaluate associations between lead measures and both uric acid and renal function as outcomes, in separate models. Covariate selection for regression models of uric acid as the outcome used *a priori* variables [age, sex, and body mass index (BMI; weight in kilograms divided by the square of height in meters)] in modeling that initially included other biologically relevant variables in separate models. Variables with *p*-values < 0.1 were then modeled together, and those with significant *p*-values in the combined model were retained. The additional covariates assessed included diabetes and hypertension (both based on participant report of physician diagnosis), use of analgesics (based on questionnaire data on medication use), work status (current vs. former lead worker), systolic and diastolic blood pressure, renal function (BUN, serum creatinine, measured creatinine clearance, and calculated creatinine clearance), tobacco use, and alcohol consumption. Serum creatinine was selected as the measure of renal function in the uric acid models because the proportion of variance explained by the model when it was included (*r*^2^ = 0.37) was the highest, compared with the other renal outcome measures. Continuous independent variables were centered at the mean or, for the effect modification models discussed below, at the tertile cut-point nearest to the mean. Covariate selection for the renal outcome models was previously reported (Weaver et al. 2003).

Finally, models with cross-product terms of the lead measures and age (age was categorized by tertiles) were evaluated, in order to assess effect modification by age on associations between the lead biomarkers and uric acid. In these models, age was also entered into the model as a centered, continuous variable, in order to avoid residual confounding.

We evaluated models for linear regression assumptions and the presence of outlying points using added variable plots (Weisberg 1985), which are graphical summaries of the relation between *Y* and a particular *X* (referred to as *X_a_* below), adjusted for all of the other covariates. Specifically, the residuals of the regression of *Y* on all of the covariates except *X_a_* are plotted on the *y*-axis. This is the part of *Y* not explained by those covariates. Next, the residuals from the regression of *X_a_* on all the other covariates are computed. This is the part of *X_a_* not explained by the other covariates. These residuals are plotted on the *x*-axis. For each plot, two lines were overlaid: the regression line, and a line determined by a scatter plot smoothing method (lowess) that calculates a locally weighted least squares estimate for each point in the scatter plot (Cleveland 1979). This allows an examination of the data for outliers that are overly influential, as evidenced by inconsistency between the lowess and regression lines (i.e., when one or two data points with both high lead dose and uric acid move the lowess line away from the regression line, they are likely to overly influence the regression line as well). When applicable, models were repeated without outliers. Models were also assessed for collinearity through examination of variance inflation factors and conditional indices.

## Results

### Selected demographics, exposure, and health outcome measures.

Information on demographics, lead biomarkers, uric acid levels, renal function, and selected comorbid conditions is presented in Tables 1 and 2. Mean (± SD) blood, tibia, and DMSA-chelatable lead levels were 32.0 ± 15.0 μg/dL, 37.2 ± 40.4 μg/g bone mineral, and 0.768 ± 0.862 mg/g creatinine, respectively. Values for these lead measures varied over a wide range. Mean values for uric acid and renal outcomes were normal, although the range for each included several abnormal outliers.

### Lead measure associations with uric acid levels.

In linear regression modeling of uric acid levels in all 803 lead workers, after adjustment for age, sex, BMI, and alcohol use, none of the lead measures was associated (Table 3). Next, we performed regression modeling to evaluate whether age, divided into tertiles (≤36 years, 36.1–46.0 years, > 46.0 years), modified relations between the lead biomarkers and uric acid levels. In models adjusted for age, sex, BMI, and alcohol use, we found evidence of effect modification by age (Table 4, method 1). Blood and tibia lead, in separate models, were associated with uric acid in participants in the oldest age tertile. As expected, because of the biologic interrelated-ness of these variables (discussed in “Materials and Methods” and shown in Figures 1 and 2), both lead associations decreased after additional adjustment for systolic blood pressure (Table 4, method 2) and renal function (Table 4, method 3). However, blood lead remained associated with uric acid (β= 0.0156, *p* = 0.01) when these associations were modeled in the 133 oldest workers who had serum creatinine greater than the median value (0.86 mg/dL).

### Associations between uric acid levels and renal outcomes.

The six renal function measures were modeled as outcomes to evaluate whether uric acid was associated with renal function in this population of lead workers. Uric acid levels were associated in all renal outcome models except NAG (Table 5). Higher uric acid was associated with worse renal function as assessed by the clinical measures but, conversely, with lower RBP. These associations remained significant after the lead biomarkers were added into the models.

### Effect of uric acid adjustment on lead measure associations in renal function models.

Associations between the lead biomarkers and the renal outcomes, after adjustment for uric acid, were modeled in the oldest tertile of workers because the associations of lead biomarkers with uric acid were in the oldest subset and the associations between higher lead dose and worse renal function were also primarily in this group. The median age of these 266 workers was 51.1 years with a range of 46.0–64.8 years. As shown in Table 6, associations between the lead measures and NAG were unchanged after adjustment for uric acid. However, fewer associations between lead biomarkers and clinical renal outcomes remained significant (*p* ≤0.05) after adjustment for uric acid.

## Discussion

In this study, we used data from the first of three evaluations in a longitudinal study of Korean lead workers to develop hypotheses about causal pathways among lead biomarkers, uric acid, renal function, and blood pressure. First, we evaluated associations of three lead dose biomarkers with uric acid, with and without control for blood pressure and renal function, while controlling for other covariates (Figure 2A). Next, we evaluated associations between uric acid and six renal function outcomes, with and without control for lead, while adjusting for blood pressure and other covariates (Figure 2B). Finally, we examined the effect of uric acid adjustment on associations between the lead biomarkers and renal outcomes, while controlling for other covariates, including blood pressure (Figure 2C).

Blood and tibia lead associations with uric acid were observed in participants in the oldest age tertile, after adjustment for age, sex, BMI, and alcohol ingestion. These associations were diminished after adjustment for blood pressure and renal function, although blood lead remained significantly associated with uric acid in the 133 oldest workers who had serum creatinine greater than the median. Next, uric acid was significantly associated with all renal function outcomes except NAG. Lastly, after adjustment for uric acid, fewer associations between lead biomarkers and the clinical renal outcomes remained significant (*p* ≤0.05).

It has been recognized for many years that individuals who have been heavily exposed to lead are at increased risk for both gout and renal disease (Batuman 1993; Shadick et al. 2000). In high-level lead exposure, urate clearance is decreased to a greater extent than can be explained by decreased glomerular filtration alone (Emmerson and Ravenscroft 1975). A defect in tubular secretion of urate is thought to be the primary factor involved (Ball and Sorensen 1969; Emmerson 1965; Emmerson and Ravenscroft 1975), although excessive tubular reabsorption (Emmerson et al. 1971) and extrarenal mechanisms such as lead effects on porphyrin metabolism (Emmerson and Ravenscroft 1975) have also been considered. Associations between lead measures and uric acid have been examined in populations encompassing a wide range of lead doses (Table 7). Relations between lead dose and gout or uric acid have also been studied in various patient populations. Increased EDTA-chelatable lead burdens have been reported in patients who have both gout and renal disease compared with other groups such as patients with gout alone or with renal disease of known non-lead-related etiology (Batuman 1993; Miranda-Carus et al. 1997; Sanchez-Fructuoso et al. 1996). Lin et al. (2001) measured blood lead and EDTA-chelatable lead in 67 patients with chronic renal insufficiency and gout and 34 patients with chronic renal insufficiency only. Mean blood lead levels were similar in the two groups (5.4 and 4.4 μg/dL, respectively), but mean EDTA-chelatable lead levels (138.1 and 64.2 μg/72 hr, respectively) were significantly (*p* < 0.01) different. All four uric acid measures were associated with EDTA-chelatable lead after adjustment for age, sex, BMI, daily protein intake, and creatinine clearance. Next, 30 participants with chronic renal insufficiency, gout, and EDTA-chelatable lead levels between 80.2 and 361 μg/72 hr were randomized to either a treatment group receiving 1 g EDTA per week for 4 weeks (*n* = 20) or a control group who received glucose in normal saline infusions. The two groups had similar uric acid, renal function, and lead measures prechelation. In the treated group, mean EDTA-chelatable lead declined from 159.2 to Each renal outcome was modeled separately. Regression results from each model are presented only for the association of uric acid with the renal outcome. BUN, serum creatinine, measured creatinine clearance, and calculated creatinine clearance models were adjusted for age, sex, BMI, current/former worker status, and hypertension. NAG and RBP models were adjusted for age, sex, BMI, systolic blood pressure, current/former worker status, alcohol ingestion, and diabetes. 41 μg/72 hr; mean serum urate decreased from 10.2 to 8.6 mg/dL (*p* = 0.02 for percent change, compared with the control group), and mean urate clearance increased from 2.7 to 4.2 mL/min (*p* < 0.01 for percent change, compared with the control group). Mean creatinine clearance also increased from 50.8 to 54.2 mL/min (*p* = 0.06 for percent change, compared with the control group). Similar uric acid findings, including results from chelation, were noted in a population of 111 participants with normal renal function, of whom 27 had gout (Lin et al. 2002).

The data discussed above and presented in Table 7 are generally consistent with the premise that in young, otherwise healthy workers, a higher lead dose, such as mean blood lead level > 50–60 μg/dL [or perhaps higher, because neither Wang et al. (2002) nor Ehrlich et al. (1998) adjusted for blood pressure or renal function], is required before associations with uric acid are present. However, in studies that include participants with other risk factors for elevated uric acid, such as older age or comorbid conditions, lower lead levels are associated with increases in uric acid.

High levels of uric acid are known to be nephrotoxic; however, controversy exists as to whether observed relations between lower levels of uric acid and renal dysfunction are causal or due to confounding. Recently, a rodent model of hyperuricemia was developed (Mazzali et al. 2001a). As noted in the introductory remarks, a range of adverse renal and vascular outcomes, similar to those noted in humans with primary hypertension (Mazzali et al. 2002) and/or renal dysfunction (Nakagawa et al. 2003), was observed in these rats. In humans, uric acid was found to be associated with reduced renal blood flow and increased renal vascular resistance in patients with primary hypertension (Messerli et al. 1980). Thus, uric acid may be nephrotoxic at lower levels than previously recognized, as opposed to being simply a marker for other renal risk factors.

Many mechanisms for the adverse affect of lead on the kidneys, either directly or through the vascular system, have been proposed (Nolan and Shaikh 1992; Sanchez-Fructuoso et al. 2002; Vaziri 2002). One mechanism not commonly considered in low to moderate lead exposure is increased uric acid. However, there are a number of similarities between the renal and vascular effects reported from low-level uric acid and those from lead exposure. Tubulointerstitial fibrosis, a classic (although nonspecific) finding in lead exposure, has been observed in the uric acid model in the absence of the urate crystals that are commonly seen in this pathology at higher levels of hyperuricemia (Mazzali et al. 2001a). Glomerular hypertrophy was reported in hyperuricemic rodents (Nakagawa et al. 2003), and Inglis et al. (1978) reported this in adults who survived childhood lead poisoning. Afferent renal arterial thickening has also been observed in hyperuricemic rats (Mazzali et al. 2002). Renal vascular disease in lead-exposed humans has been reported in several case series (Inglis et al. 1978; Morgan et al. 1966; Wedeen et al. 1975). Sanchez-Fructuoso et al. (2002) recently reported hypertrophy of the medium and small renal arteries and arterioles in rats whose blood lead levels ranged from 52.9 to 33.2 μg/dL at day 90 (when lead ingestion ceased). However, these vascular abnormalities were not observed in rats whose lead exposures, over a 12-month period, were either lower (blood lead levels ~ 20–30 μg/dL) (Khalil-Manesh et al. 1993) or much higher (blood lead levels of 45.5–125.4 μg/dL, averaged over a 12-month period) (Khalil-Manesh et al. 1992). Uric acid was not measured in these rodent studies; however, Goyer (1971) reported hyperuricemia that was not thought to be related to extent of renal insufficiency in lead-exposed rats, which suggests that lead may be one of the exposures that does increase uric acid in rats despite the presence of the uricase enzyme. Mazzali et al. (2001a) reported that increased systolic blood pressure was correlated with serum uric acid. Increased systolic blood pressure was associated with lead dose in the same Korean lead worker population studied in this report (Lee et al. 2001); similar associations have also been reported in other populations (Sharp et al. 1987). Increased juxtaglomerular renin staining was present in the uric acid model (Mazzali et al. 2001a). Data suggest that lead exposure also increases renin; this effect may vary with length of exposure. Several reviews have concluded that renin is increased with short- to moderate-term lead exposure in both animals and humans but is normal or decreased with prolonged exposure (Gonick and Behari 2002; Sharp et al. 1987; Vander 1988). Decreased neuronal nitric oxide synthase expression in the macula densa was reported in rodents in the uric acid model (Mazzali et al. 2001a). In contrast, the effect of lead on NO does not involve decreased NO synthase expression (Vaziri 2002). In fact, just the opposite occurs because lead exposure generates oxidants that deplete NO, and NO synthase expression is up-regulated in response.

## Conclusion

Our data suggest that, at the moderate levels of lead exposure present in our population, older workers comprise a susceptible population for increased uric acid. This is consistent with the published literature, as noted above. The impact of adjustment for renal function and blood pressure suggests that the effect of lead on uric acid may be mediated through these pathways (Figure 2A). However, because blood lead remained associated with uric acid in our most susceptible group (the oldest workers who had the greatest renal dysfunction), even after adjustment for renal function and blood pressure, mechanisms other than decreased glomerular filtration, such as decreased tubular secretion or even extrarenal mechanisms, may be involved at these exposure levels. Because our data [and those of others (Shadick et al. 2000)] suggest an effect of lead on uric acid beyond that due to renal dysfunction alone, and because uric acid was associated with adverse renal outcomes and resulted in reduced significance of lead biomarker associations in our population, uric acid may be one mechanism through which lead is nephrotoxic. However, this is not the only mechanism for lead-related nephrotoxicity. In our data, the association between blood lead and serum creatinine remained significant (*p* < 0.05) even after adjustment for uric acid. Associations between lead dose and NAG were unchanged, and uric acid was inversely associated with RBP. The effects of lead and uric acid on the NO system are also different. Thus, other mechanisms must be involved.

Conclusions regarding causality in this study must be limited because it is cross-sectional. An additional limitation is that we were not able to adjust for the use of medications that influence uric acid because Koreans are not routinely provided with the names of their medications. However, few participants reported any prescription medication use. Our results do suggest that further evaluation of relations among the lead dose biomarkers, uric acid, and renal function in our longitudinal data set would be of value. This is particularly true because these mechanistic relations may be clinically important. EDTA chelation has been reported to improve both renal function and urate clearance in patients with renal insufficiency and gout, even when EDTA-chelatable lead body burdens were quite low (Lin et al. 2001). If this work is replicated in other populations and low-level uric acid is found to be nephrotoxic, uric acid should also be monitored in patients who are in the early stages of diseases such as early chronic renal insufficiency and whose lead body burdens are amenable to chelation.

## Figures and Tables

**Table 1:** Selected demographic, exposure, and health outcome measures (categorical variables) of 803 current and former lead workers in South Korea.

Characteristic	No. (%)
Sex	
Male	639 (79.6)
Female	164 (20.4)
Work status	
Current lead worker	709 (88.3)
Former lead worker	94 (11.7)
Diabetes	6 (0.8)
Hypertension	58 (7.2)
Regular analgesic use	16 (2.0)
Alcohol use	
Never	233 (29.1)
Current use	521 (65.0)
Past use	48 (6.0)
Tobacco use	
Never	255 (31.8)
Current use	458 (57.1)
Past use	89 (11.1)

**Table 2:** Selected demographic, exposure, and health outcome measures (continuous variables) of 803 current and former lead workers in South Korea.

Health outcome	Mean ± SD	Range
Age (years)	40.4 ± 10.1	17.8–64.8
BMI (kg/m^2^)	23.0 ± 3.0	15.7–34.2
Systolic blood pressure (mm Hg)	123.2 ± 16.3	83.7–215.3
Diastolic blood pressure (mm Hg)	75.7 ± 12.0	36.0–126.7
Blood lead (μg/dL)	32.0 ± 15.0	4.3–85.7
Tibia lead (μg Pb/g bone mineral)	37.2 ± 40.4	^−^7.4–337.6
DMSA-chelatable lead (mg Pb/g creatinine)*^a^*	0.768 ± 0.862	0.02–8.98
Lead job duration (years)	8.2 ± 6.5	< 1–36.2
Uric acid (mg/dL)	4.8 ± 1.2	1.4–12.3
BUN (mg/dL)	14.4 ± 3.7	6–32.2
Serum creatinine (mg/dL)	0.90 ± 0.16	0.48–2.5
Measured creatinine clearance (mL/min)*^a^*	114.7 ± 33.6	11.8–338.9
Calculated creatinine clearance (mL/min)	94.7 ± 20.7	41.1–184.5
NAG (μmol/hr/g creatinine)	215.3 ± 188.5	13.8–2577.0
RBP (μg/g creatinine)	63.6 ± 190.6	5.2–4658.7

a *n* = 787.

**Table 3:** Linear regression models to evaluate associations of lead dose biomarkers with uric acid levels (*n* = 803).

Model	Lead variable	β-coefficient	SE β	*p*-Value	Model *r*^2^
1	Tibia lead (μg Pb/g bone mineral)	−0.0005	0.0010	0.62	0.32
2	Blood lead (μg/dL)	0.0027	0.0027	0.32	0.31
3	DMSA-chelatable lead (μg Pb/g creatinine)	0.0259	0.0431	0.55	0.31

Uric acid was modeled separately as the outcome, with one of the three lead biomarkers included per model. Regression results from each model are presented only for the association of the lead biomarker with uric acid. Models were also adjusted for age, sex, BMI, and alcohol use.

**Table 4:** Linear regression models to evaluate effect modification by age in tertiles on associations of blood and tibia lead with uric acid in all lead workers, with outliers removed (method 1), and with additional control for systolic blood pressure (method 2) and serum creatinine (model 3) (*n* = 803).

	Method 1			Method 2			Method 3		
Variable	β-coefficient	SE β	*p*-Value	β-coefficient	SE β	*p*-Value	β-coefficient	SE β	*p*-Value
Blood lead model									
Intercept	4.9217	0.0757	< 0.01	4.9350	0.0759	< 0.01	4.8528	0.0736	< 0.01
Age (years)	−0.0182	0.0039	< 0.01	−0.0199	0.0040	< 0.01	−0.0210	0.0039	< 0.01
Systolic blood pressure (mm Hg)	—	—	—	0.0047	0.0023	0.04	0.0046	0.0022	0.04
Serum creatinine (mg/dL)	—	—	—	—	—	—	2.1830	0.2666	< 0.01
Blood lead (μg/dL)	0.0111	0.0041	< 0.01	0.0105	0.0041	0.01	0.0071	0.0039	0.07
Blood lead × age category 2	−0.0109	0.0057	0.05	−0.0107	0.0056	0.06	−0.0063	0.0054	0.25
Blood lead × age category 1	−0.0150	0.0058	0.01	−0.0148	0.0058	0.01	−0.0107	0.0056	0.06
Tibia lead model									
Intercept	4.8932	0.0749	< 0.01	4.9087	0.0750	< 0.01	4.8430	0.0735	< 0.01
Age (years)	−0.0155	0.0039	< 0.01	−0.0174	0.0040	< 0.01	−0.0184	0.0038	< 0.01
Systolic blood pressure (mm Hg)	—	—	—	0.0052	0.0022	0.02	0.0048	0.0022	0.03
Serum creatinine (mg/dL)	—	—	—	—	—	—	2.1808	0.3189	< 0.01
Tibia lead (μg Pb/g bone mineral)	0.0036	0.0018	0.04	0.0031	0.0018	0.08	0.0019	0.0017	0.28
Tibia lead × age category 2	−0.0057	0.0028	0.04	−0.0053	0.0028	0.06	−0.0019	0.0028	0.49
Tibia lead × age category 1	−0.0071	0.0029	0.02	−0.0067	0.0029	0.02	−0.0044	0.0029	0.13

—, Variable not included in method. Models were also adjusted for sex, BMI, and alcohol use. The oldest age tertile is the reference category. Slopes in the middle (age category 2) and youngest (age category 1) age categories are obtained by adding their respective β-coefficients (of the cross-product term for age × lead) to the β-coefficient of the reference category (oldest age group). *p*-Values for the cross-product terms reflect the statistical significance of the difference between the slopes of the regression line in that age category and the regression line for the oldest age group.

**Table 5:** Linear regression models to evaluate associations of uric acid with renal outcomes while controlling for covariates (*n* = 803).

Model	Renal function outcome	Uric acid β-coefficient	SE β	*p*-Value
1	BUN (mg/dL)	0.4186	0.1246	< 0.01
2	Serum creatinine (mg/dL)	0.0267	0.0038	< 0.01
3	Measured creatinine clearance (mL/min)	−2.5300	0.9791	0.01
4	Calculated creatinine clearance (mL/min)	−2.1700	0.4662	< 0.01
5	ln NAG [ln (μmol/hr/g creatinine)]	−0.0262	0.0210	0.21
6	ln RBP [ln (μg/g creatinine)]	−0.1067	0.0254	< 0.01

**Table 6:** Linear regression models to evaluate associations of lead dose biomarkers and uric acid levels with renal outcomes in 266 lead workers in the oldest tertile of age.

	Method 1 (lead biomarker models)			Method 2 (uric acid models)			Method 3 (combined models)		
Independent variables	βcoefficient	SE β	*p*-Value	βcoefficient	SE β	*p*-Value	βcoefficient	SE β	*p*-Value
BUN (mg/dL) models									
Blood lead (μg/dL)	0.0352	0.0183	0.05	—	—	—	0.0293	0.0185	0.11
Uric acid (mg/dL)	—	—	—	0.4663	0.2307	0.04	0.3963	0.2343	0.09
Serum creatinine (mg/dL) models									
Blood lead (μg/dL)	0.0016	0.0006	< 0.01	—	—	—	0.0012	0.0006	0.03
Uric acid (mg/dL)	—	—	—	0.0245	0.0072	< 0.01	0.0215	0.0073	< 0.01
Tibia lead (μg Pb/g bone mineral)	0.0004	0.0002	0.03	—	—	—	0.0003	0.0002	0.06
Uric acid (mg/dL)	—	—	—	0.0246	0.0072	< 0.01	0.0233	0.0072	< 0.01
Measured creatinine clearance (mL/min) models									
Blood lead (μg/dL)	0.1187	0.1177	0.31	—	—	—	0.1697	0.1198	0.16
Uric acid (mg/dL)	—	—	—	−2.4871	1.4456	0.09	−2.9352	1.4769	0.05
Calculated creatinine clearance (mL/min) models									
Blood lead (μg/dL)	−0.1221	0.0594	0.04	—	—	—	−0.0950	0.0600	0.11
Uric acid (mg/dL)	—	—	—	−2.0384	0.7487	< 0.01	−1.8095	0.7604	0.02
ln NAG [ln (μmol/hr/g creatinine)] models									
Blood lead (μg/dL)	0.0089	0.0028	< 0.01	—	—	—	0.0092	0.0028	< 0.01
Uric acid (mg/dL)	—	—	—	−0.0115	0.0364	0.76	−0.0289	0.0361	0.42
Tibia lead (μg Pb/g bone mineral)	0.0023	0.0008	< 0.01	—	—	—	0.0023	0.0008	< 0.01
Uric acid (mg/dL)	—	—	—	−0.0070	0.0366	0.85	−0.0094	0.036	0.80
DMSA-chelatable lead (mg Pb/g creatinine)	0.1931	0.0511	< 0.01	—	—	—	0.1944	0.0512	< 0.01
Uric acid (mg/dL)	—	—	—	−0.0182	0.0373	0.63	−0.0235	0.0363	0.52

BUN, serum creatinine, measured creatinine clearance, and calculated creatinine clearance models were also adjusted for age, sex, BMI, current/former worker status, and hypertension. NAG and RBP models were adjusted for age, sex, BMI, systolic blood pressure, current/former worker status, alcohol ingestion, and diabetes. Only models in which *p* ≤0.05 for the lead variable without uric acid adjustment are shown, with the exception of the measured creatinine clearance model; this model is included because the *p*-value for the β-coefficient of the uric acid variable decreased to ≤0.05 after adjustment for blood lead.

**Table 7:** Summary of selected publications*^a^* that have evaluated lead measure associations with uric acid.

Study	No.	Mean age (years)	Mean blood or bone lead*^b^*	Association	*p*-Value of lead measure	Covariates controlled for	Comments
Wang et al. 2002	229	65%	67.7 μg/dL, males	10 μg/dL increase in blood lead associated with a 0.085 mg/dL increase in uric acid	0.02	Sex and body weight	Alcohol apparently not significant
		< 40	48.6 μg/dL, females				
Ehrlich et al. 1998	382	41	53.5 μg/dL	Current and historical blood lead in quintiles associated with uric acid	≤0.01 for trend	Age, height, and weight	Tibia lead measured on a random sample of 40 participants
			69.7 μg/g				
Roels et al. 1994	76*^c^*	44	43.0 μg/dL; 66 μg/g	Continuous lead measures (workers plus controls) with uric acid	NS	Not reported	
	68*^d^*	43	14.1 μg/dL; 21 μg/g				
Baker et al. 1981	318	36*^e^*	22.4 μg/dL*^e^*	Continuous blood lead with uric acid	NS	Age	
		37*^f^*	24.0 μg/dL*^f^*				
Smith et al. 1995	691	48	7.8 μg/dL	Continuous blood lead with uric acid	NS	Age, alcohol, ALAD	
Shadick et al. 2000	777	67	5.9 μg/dL	Blood lead and uric acid	0.1	Age, BMI, diastolic blood pressure, alcohol, serum creatinine	Normative Aging Study
			30.2 μg/g patella	Patella lead and uric acid	0.02		
			20.8 μg/g tibia	Tibia lead and uric acid	0.06		

Abbreviations: ALAD, δ-aminolevulinic acid dehydrase; NS, not significant.

a Based on sample size and extent of statistical analysis.

b μg/g indicates tibia lead per bone mineral unless noted as patella.

c Lead workers.

d Controls.

e Rural residence.

f Urban residence.
